# Prognostic value of ^18^F-fluorodeoxyglucose positron emission tomography in patients with small hepatocellular carcinoma treated by radiofrequency ablation

**DOI:** 10.1186/s40644-020-00356-5

**Published:** 2020-10-19

**Authors:** Yoshiyuki Ida, Hideyuki Tamai, Naoki Shingaki, Ryo Shimizu, Shuya Maeshima, Takao Maekita, Mikitaka Iguchi, Masaki Terada, Masayuki Kitano

**Affiliations:** 1 Second Department of Internal Medicine, Wakayama Medical University, 811-1 Kimiidera, Wakayama, 641-0012 Japan; 2 Department of Hepatology, Wakayama Rosai Hospital, 93-1 Kinomoto, Wakayama, 640-8505 Japan; 3 Wakayama Minami Radiology Clinic, 870-2 Kimiidera, Wakayama, 641-0012 Japan

**Keywords:** 18F-fluorodeoxyglucose positron emission tomography, Hepatocellular carcinoma, Radiofrequency ablation

## Abstract

**Background:**

^18^F-fluorodeoxyglucose (^18^F-FDG) uptake in hepatocellular carcinoma (HCC) is significantly associated with early recurrence and survival after curative surgical resection. However, there are no reports regarding the relationship between ^18^F-FDG uptake and outcomes after radiofrequency ablation (RFA). A prospective cohort study was conducted to evaluate the prognostic value of ^18^F-FDG positron emission tomography (PET) in HCC patients after RFA.

**Methods:**

A total of 121 consecutive patients with primary HCC (≤3 tumors, of diameter ≤ 3 cm) without vascular invasion on imaging were examined by ^18^F-FDG-PET computed tomography prior to RFA. An HCC with a component of ^18^F-FDG uptake visibly stronger than that of surrounding liver was defined as ^18^F-FDG-PET positive.

**Results:**

The median follow-up period was 1267 days. There were 110 ^18^F-FDG-PET negative and 11 positive tumors. The cumulative 1-year recurrence rates in the ^18^F-FDG negative and positive groups were 30 and 64% (*P =* 0.017), respectively, and cumulative 1-year metastatic recurrence rates were 6 and 36% (*P <* 0.001), respectively. The cumulative 5-year survival rates were 88 and 22% (*P <* 0.001), respectively. Multivariate analysis revealed ^18^F-FDG-PET positivity and tumor size as independent factors related to metastatic recurrence and survival after RFA.

**Conclusions:**

^18^F-FDG-PET positivity was significantly associated with outcomes after RFA. RFA should not be readily selected as the first-line treatment for small HCC that includes a component of visually strong ^18^F-FDG uptake.

## Background

Radiofrequency ablation (RFA) is established as the standard of care for patients with small hepatocellular carcinoma (HCC) unsuitable for surgical resection. Clinical practice guidelines for HCC state that RFA is indicated for ≤3 tumors, of diameter ≤ 3 cm [1–3]. However, some reports have indicated that along with the pathologic differentiation that occurs in advanced HCC, there is an increased incidence of microscopic vascular invasion and intrahepatic metastasis even in small HCC [4–7], and the prognosis after RFA becomes poor [8]. In addition, numerous studies have reported an association between RFA and severe problems such as intrahepatic dissemination [9–11], aggressive recurrence with vascular invasion [12–15], and seeding [16]. Because the risks of these types of critical recurrence after RFA are related to tumor characteristics at the time of ablation (e.g., poor differentiation and vascular invasion), the histological differentiation grade should be assessed in determining the optimal treatment plan, even in patients with small HCC. However, tumor biopsy has limitations related to tumor location, sampling error, and the risk of complications such as bleeding and tumor seeding [17].

^18^F-FDG positron emission tomography (PET) is already in common use in screening for various cancers, including for lung and breast cancer. However, as the sensitivity of ^18^F-FDG-PET for detecting HCC is lower than that of contrast-enhanced computed tomography (CT) and magnetic resonance imaging (MRI), ^18^F-FDG-PET is therefore no longer recommended as a standard imaging modality for the early diagnosis of HCC [3]. However, it has been reported that ^18^F-FDG-PET uptake is associated with poor prognosis after surgical resection [18, 19], and that it can predict vascular invasion and recurrence in HCC patients before liver transplantation [20, 21]. If there is a relationship also between ^18^F-FDG-PET uptake and outcomes after RFA, ^18^F-FDG-PET would be useful when considering the optimal and safe treatment strategy for small HCC. The aim of this prospective cohort study was to clarify whether ^18^F-FDG-PET uptake is associated with outcomes after RFA for small HCC.

## Methods

### Patients

Included in the study were adult patients with primarily diagnosed HCCs who underwent RFA. The exclusion criteria were any of the following: 1) four tumors or more, 2) tumor diameter > 3 cm, 3) severe decompensated cirrhosis (Child–Pugh class C). A total of 121 consecutive patients with initially diagnosed HCCs had undergone ^18^F-FDG-PET CT within 4 weeks before RFA between May 2008 and February 2013. Any two of contrast CT, dynamic MRI, or contrast ultrasonography were performed for the differential diagnosis of liver tumor. HCC was diagnosed based on “typical imaging finding,” which is typically defined as a nodule that is visualized as a high signal intensity area in the arterial phase and relatively low signal intensity area in the venous phase [22]. Prior to RFA, the following were recorded in all patients: tumor diameter; etiology of hepatitis; Child–Pugh classification; platelet count; serum alanine aminotransferase (ALT) level; levels of the tumor markers alpha-fetoprotein (AFP), *Lens culinaris* agglutinin-reactive alpha-fetoprotein (AFP-L3), des-gamma-carboxy prothrombin (DCP); and serum fibrosis markers (type IV collagen 7S and hyaluronic acid). This prospective observational study was approved by our ethics committee and conformed to the provisions of the Helsinki Declaration. Written informed consent was obtained from all patients.

### ^18^F-FDG-PET imaging protocol

All patients were imaged prior to RFA by a whole-body PET scanner (Eminence-B, Shimadzu, Kyoto, Japan) with axial resolution of 3.9 mm (full-width at half maximum) and a 20-cm field of view (z axis). Prior to scanning, patients fasted for at least 5 h, and their blood glucose level at the time of FDG injection was < 150 mg/dl. Each patient received intravenous injection of approximately 2.6 MBq ^18^F-FDG per kg of body weight, and was scanned from the head to the upper thigh (slice thickness, 8 mm) 50 min after injection. FDG images were corrected for attenuation with a cesium external source.

### Image analysis

**Fig. 1:**
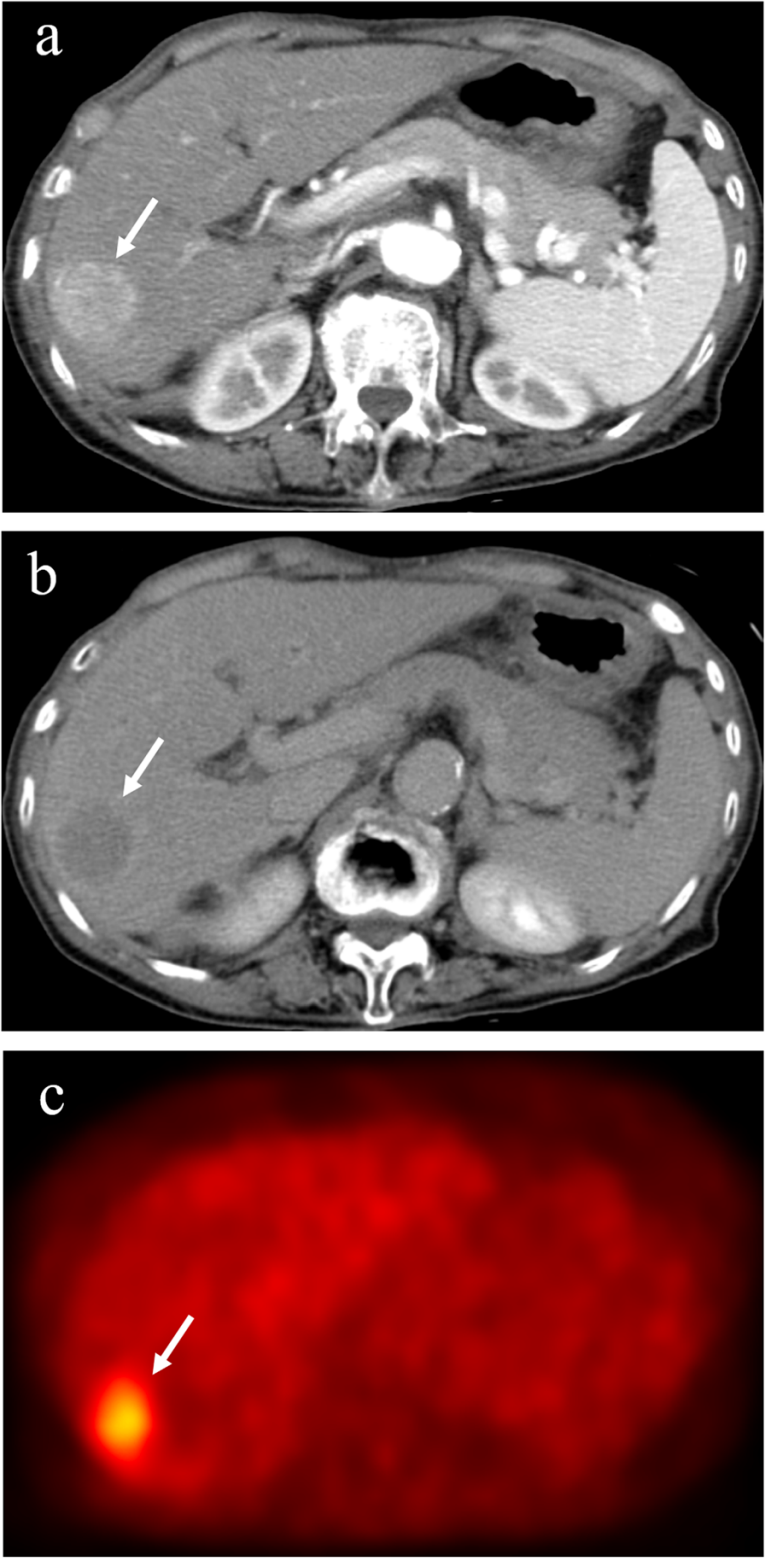
Hepatocellular carcinoma with positive ^18^F-fluorodeoxyglucose uptake on positron emission tomography. The hepatocellular carcinoma (diameter, 3.0 cm) in segment 5 exhibits staining during the arterial phase of contrast computed tomography (**a**) and washout during the equilibrium phase (**b**). The tumor has higher ^18^F-fluorodeoxyglucose uptake than that of surrounding liver on positron emission tomography (**c**). Arrows indicate the tumor. ^18^F-FDG, ^18^F-fluorodeoxyglucose; PET, positron emission tomography

**Fig. 2:**
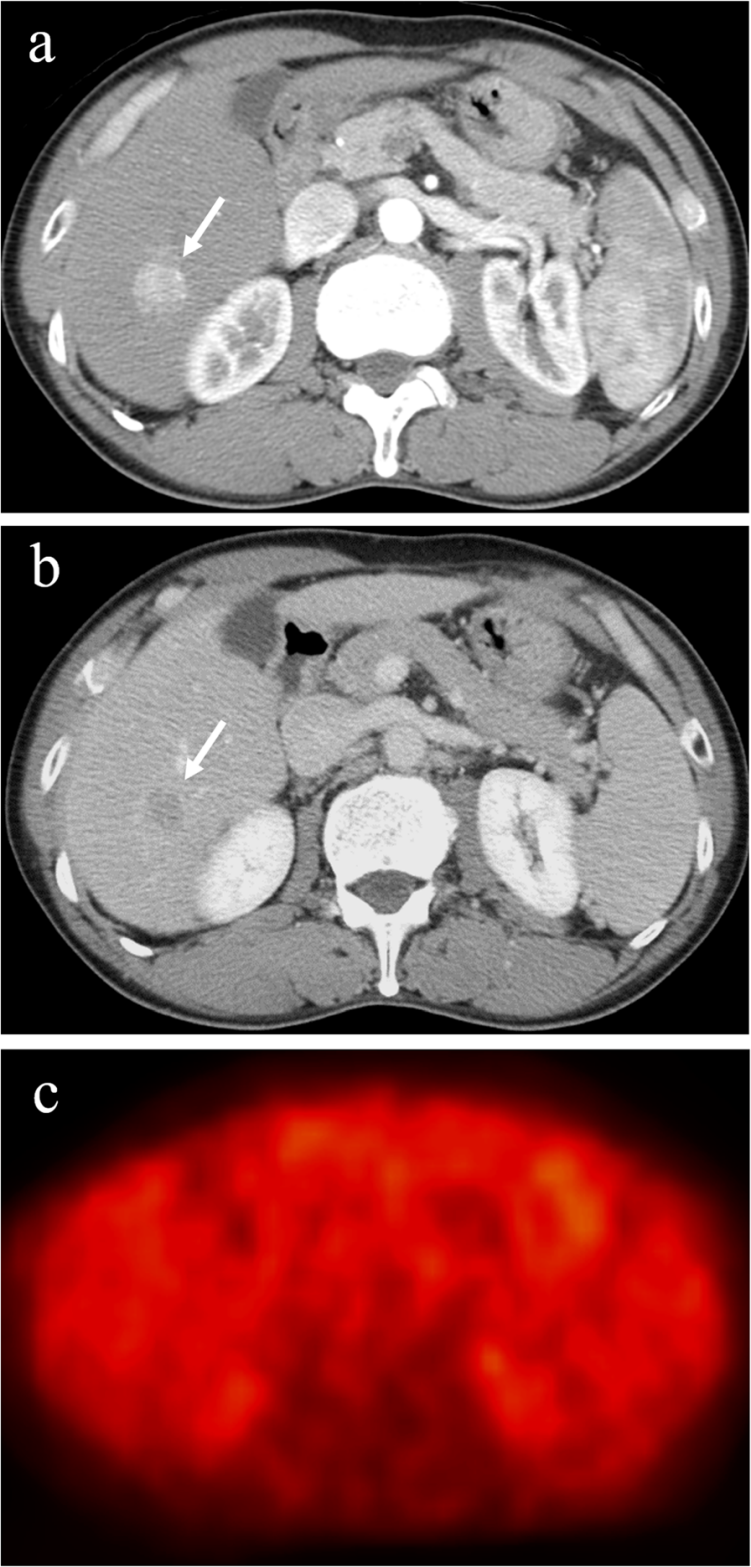
Hepatocellular carcinoma with negative ^18^F-fluorodeoxyglucose uptake on positron emission tomography. The hepatocellular carcinoma (diameter, 2.0 cm) in segment 6 exhibits staining during the arterial phase of contrast computed tomography (**a**) and washout during the equilibrium phase (**b**). The tumor has equal ^18^F-fluorodeoxyglucose uptake to that of surrounding liver on positron emission tomography (**c**). Arrows indicate the tumor

The ^18^F-FDG-PET images were independently analyzed with reference to the contrast CT or dynamic MRI images by one experienced radiologist and one experienced hepatologist, each with > 20 years of experience in liver imaging. Any disagreements in interpretation were resolved by consensus. The degree of ^18^F-FDG uptake in a nodule seen on ^18^F-FDG-PET was visually compared with that in the surrounding liver. Tumors with stronger ^18^F-FDG uptake, as a whole or partially, in comparison with the surrounding liver were termed PET positive (Fig. 1), and those with ^18^F-FDG uptake equal to the surrounding liver were termed PET negative (Fig. 2). In the case of multiple HCCs, ^18^F-FDG uptake in the largest tumor was analyzed.

### RFA technique

Percutaneous RFA was performed in all patients, using the Cool-tip RF system (COVIDEN, Boulder, CO, USA) under ultrasound guidance. An artificial pleural effusion or artificial ascites was produced, using saline when necessary. Impedance control mode was used with a 17-G cooled-tip electrode with a 2- or 3-cm exposed tip. Ablation was started at 40 W for the 2-cm exposed tip and at 60 W for the 3-cm exposed tip, and power was increased at a rate of 10 W/min. When a rapid increase in impedance occurred, the output was automatically stopped and ablation was restarted after a short time at an output 10 W lower. The duration of a single ablation was 6 min for the 2-cm electrode and 12 min for the 3-cm electrode. After RF exposure, the temperature of the needle tip was measured. When the temperature was < 65℃, additional ablation was performed.

### Assessment of response and follow up

Treatment response was assessed by contrast CT or MRI at 1–3 days after the final session. Complete response was defined as no enhancement in the entire lesion on imaging, with a safety margin. Additional ablation was performed until complete ablation was confirmed in each nodule. All patients were followed up on an outpatient basis every 3–4 months, including contrast CT or MRI and measurement of tumor marker levels. In the case of extrahepatic metastasis, diagnosis was performed by ^18^F-FDG-PET CT or biopsy. As imaging methods cannot distinguish whether multiple intrahepatic recurrences are metastatic or multicentric non-concurrent primary lesions, intrahepatic metastatic recurrence was defined as the presence of at least three hypervascular intrahepatic recurrences, as stated as the criteria for metastatic recurrences attributed to advanced stage cancer as an indication for RFA (≤3 tumors, of diameter ≤ 3 cm).

### Statistical analysis

Values are expressed as the median (range). The Mann–Whitney U test was used to analyze continuous variables, and Fisher’s exact test or the χ^2^ test was used to analyze categorical variables. Cumulative recurrence-free survival rates, cumulative metastatic recurrence-free survival rates, and cumulative survival rates according to the ^18^F-FDG-PET positivity classification were calculated using the Kaplan–Meier method and compared using the log-rank test. Univariate and multivariate analyses for factors related to recurrence, including metastatic recurrence, and survival related to HCC were performed using a Cox proportional hazards regression model. The results are expressed as hazard ratios (HRs) with 95% confidence intervals (CIs). Values of *p <* 0.05 were considered significant. All analyses were performed using the SPSS 21.0 software package (SPSS, Inc., Chicago, IL, USA).

## Results

### Patient characteristics

The median follow-up period was 1267 days. Table 1 lists the characteristics of the enrolled patients.

**Table 1:** Patients’ baseline characteristics (*n* = 121)

Age (years)	69 (45–87)
Sex (male/female)	73/48
Etiology (HCV/non-HCV)	91/30
Fibrosis stage (F0/1/2/3/4)	2/0/12/30/77
Tumor size (mm)	18 (8–30)
AFP (ng/mL)	18.0 (1.8–1594.5)
AFP-L3 (%)	7.4 (0.0–80.0)
DCP (mAU/mL)	64 (5–9489)

*HCV* hepatitis C virus, *AFP* alpha-fetoprotein, *AFP-L3 Lens culinaris* agglutinin-reactive alpha-fetoprotein, *DCP* Des-gamma-carboxyprothrombin. Data are expressed as medians (range)

### Comparison of baseline characteristics between ^18^F-FDG-PET negative and ^18^F-FDG-PET positive groups

Table 2 shows a comparison of the baseline characteristics of patients in the ^18^F-FDG-PET positive and negative groups. Tumor size, AFP, AFP-L3, and DCP values were significantly higher in the ^18^F-FDG-PET positive group than the negative group. No significant differences were seen in terms of age, sex, etiology, Child–Pugh classification, platelet count, ALT level, fibrosis markers, or number of tumors between the ^18^F-FDG-PET positive and negative groups.

**Table 2:** Comparison of baseline characteristics between the PET negative and PET positive groups

	PET negative (*n* = 110)	PET positive (*n* = 11)	***p*** value
Age (years)	69 (45–87)	76 (58–83)	0.401
Sex (male/female)	68/42	5/6	0.341
Etiology (HCV/non-HCV)	82/28	9/2	0.730
Fibrosis stage (F0–3/4)	39/71	5/6	0.526
Child–Pugh class (A/B)	87/23	10/1	0.691
Platelets (× 10^4^/μL)	9.0 (2.4–75.9)	10.4 (2.9–21.2)	0.351
ALT (IU/L)	37 (10–171)	45 (16–133)	0.339
Hyaluronic acid (ng/mL)	216.3 (23.2–1851.9)	265.5 (85.0–884.9)	0.438
Type IV collagen 7S (ng/mL)	7.7 (3.0–18.0)	7.3 (4.1–12.9)	0.487
Tumor size (mm)	18 (8–30)	25 (15–30)	0.001
Number of tumors (1/2/3)	91/14/5	8/2/1	0.413
AFP (ng/mL)	16.1 (1.8–1594.5)	112.1 (3.1–1545.8)	0.011
AFP-L3 (%)	7.3 (0–72.5)	28.9 (0–80.0)	0.011
DCP (mAU/mL)	58 (5–9489)	177 (22–2615)	0.008

*HCV* hepatitis C virus, *ALT* alanine aminotransferase, *HCC* hepatocellular carcinoma, *AFP* alpha-fetoprotein, *AFP-L3 Lens culinaris* agglutinin-reactive alpha-fetoprotein, *DCP* Des-gamma-carboxyprothrombin, *PET* positron emission tomography. Data are expressed as medians (range)

### Comparison of recurrence and metastatic recurrence after RFA between ^18^F-FDG-PET negative and ^18^F-FDG-PET positive groups

**Fig. 3:**
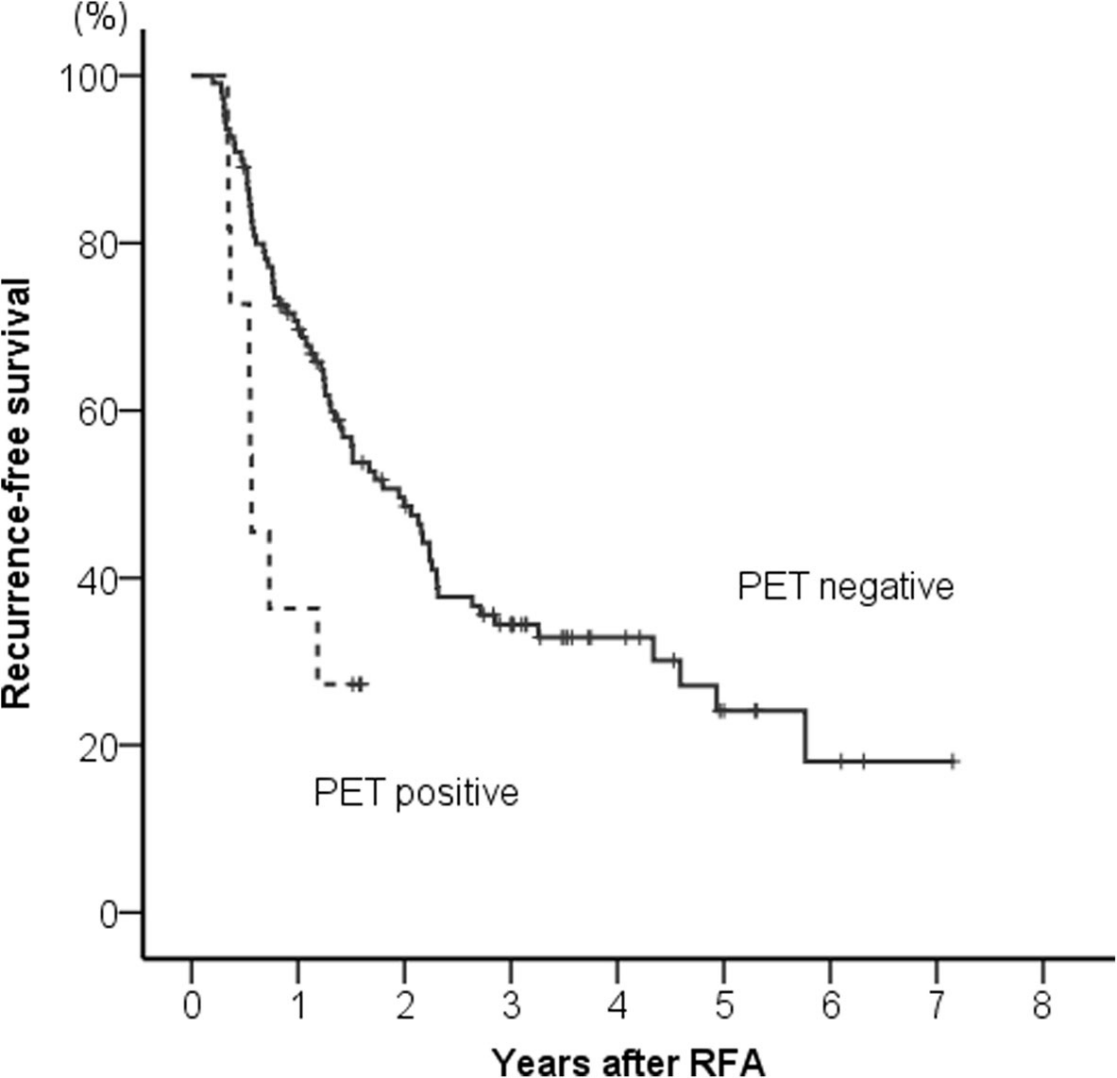
Comparison of recurrence-free survival between ^18^F-fluorodeoxyglucose positron emission tomography positive and negative groups. Recurrence-free survival was significantly shorter in the ^18^F-fluorodeoxyglucose positron emission tomography positive group than in the negative group (*p* = 0.017)

Recurrence-free survival curves according to ^18^F-FDG-PET positivity are shown in Fig. 3. Recurrence-free survival was significantly shorter in the ^18^F-FDG-PET positive group than the negative group (*p* = 0.017). The cumulative 1-year recurrence rates of the ^18^F-FDG-PET negative and positive groups were 30 and 64%, respectively.

**Fig. 4:**
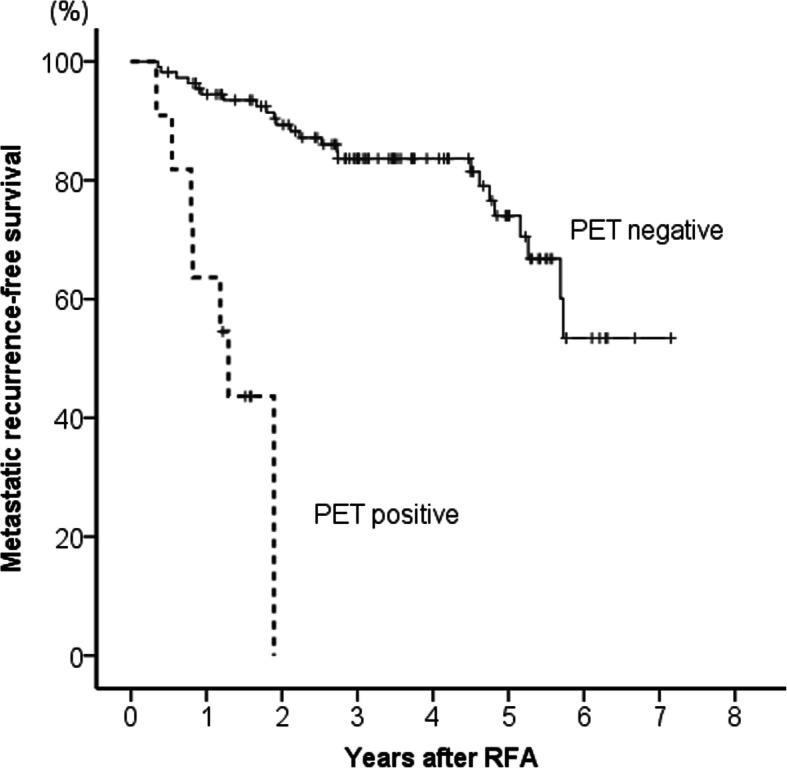
Comparison of metastatic recurrence-free survival rates between ^18^F-fluorodeoxyglucose positron emission tomography positive and negative groups. Metastatic recurrence-free survival was significantly shorter in the ^18^F-fluorodeoxyglucose positron emission tomography positive uptake group than in the negative group (*p* < 0.001)

Metastatic recurrence-free survival curves according to ^18^F-FDG-PET positivity are shown in Fig. 4. Metastatic recurrence-free survival was significantly shorter in the ^18^F-FDG-PET positive group than in the negative group (*p* < 0.001). The cumulative 1-year metastatic recurrence rates of the ^18^F-FDG-PET negative and positive groups were 6 and 36%, respectively. Metastatic recurrences occurred in 30 patients during the follow-up period, as follows: intrahepatic metastasis (*n* = 18); extrahepatic and intrahepatic metastases (*n* = 2); extrahepatic metastasis (*n* = 2); intrahepatic metastasis and portal invasion (*n* = 4); extrahepatic metastasis and portal invasion (*n* = 2); extrahepatic metastasis and hepatic vein invasion (*n* = 1); and intrahepatic metastasis, extrahepatic metastasis, and portal invasion (each *n* = 1). During the follow-up period, 48% (15/31) of patients with metastatic recurrences died from HCC.

### Comparison of survival between ^18^F-FDG-PET negative and ^18^F-FDG-PET positive groups

**Fig. 5:**
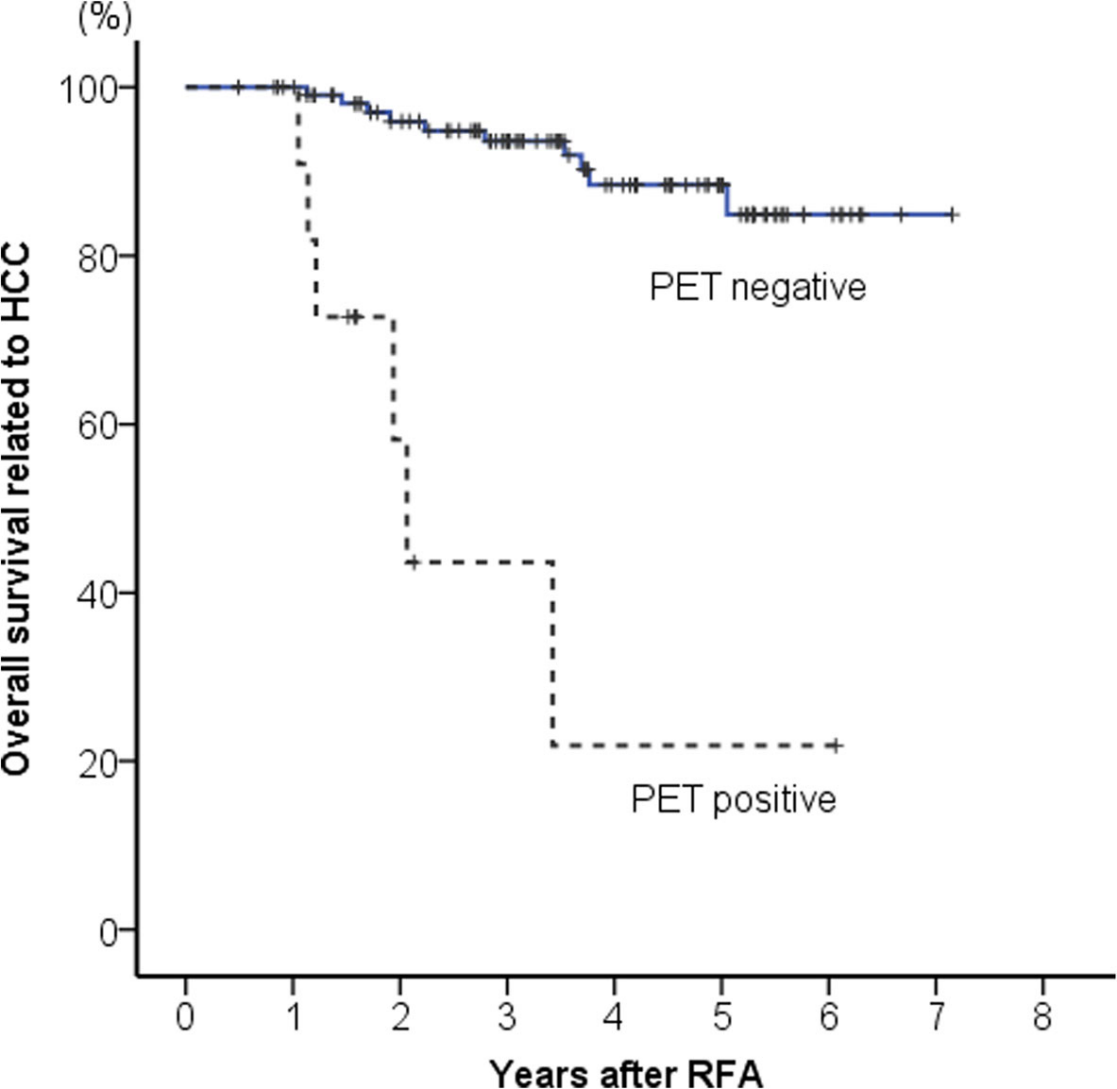
Comparison of survival between ^18^F-fluorodeoxyglucose positron emission tomography positive and negative groups. Survival was significantly shorter in the ^18^F-fluorodeoxyglucose positron emission tomography positive group than in the negative group (*p* < 0.001)

Survival curves according to ^18^F-FDG-PET positivity are shown in Fig. 5. Survival was significantly shorter in the ^18^F-FDG-PET positive group than in the negative group (*p* < 0.001). The cumulative 5-year survival rate was 88% in the ^18^F-FDG-PET negative group and 22% in the ^18^F-FDG-PET positive group.

### Univariate and multivariate analyses of factors related to recurrence

Table 3 lists the results of univariate and multivariate analyses of background variables associated with overall recurrence. Univariate analysis identified the factors of etiology (hepatitis C virus), Child–Pugh classification, ALT level, hyaluronic acid level, type IV collagen 7S level, tumor size, number of tumors, and ^18^F-FDG-PET positivity as being significantly associated with recurrence after RFA. In multivariate analysis, etiology (hepatitis C virus), Child–Pugh classification, and number of tumors were identified as independent factors.

**Table 3:** Univariate and multivariate analyses of factors related to recurrence

	Univariate analysis			Multivariate analysis		
	*p* value	HR	95%CI	*p* value	HR	95%CI
Age (years)	0.520	1.008	0.983–1.034			
Sex (female)	0.878	1.036	0.660–1.627			
Etiology (HCV)	0.004	2.426	1.334–4.411	0.006	2.438	1.293–4.599
Child–Pugh class (A)	0.007	0.492	0.294–0.823	0.042	0.524	0.281–0.976
Platelets (×10^4^/μL)	0.742	0.994	0.957–1.031			
ALT (IU/L)	0.048	1.006	1.000–1.012	0.873	1.001	0.993–1.008
Hyaluronic acid (ng/mL)	< 0.001	1.001	1.001–1.002	0.575	1.000	0.999–1.001
Type IV collagen 7S (ng/mL)	0.000	1.146	1.073–1.224	0.130	1.080	0.978–1.192
Tumor size (mm)	0.037	1.040	1.002–1.079	0.573	1.012	0.971–1.054
Number of tumors	< 0.001	2.172	1.459–3.235	0.005	1.930	1.219–3.057
AFP (ng/mL)	0.120	1.001	1.000–1.001			
AFP-L3 (> 15%)	0.084	1.725	0.929–3.205			
DCP (mAU/mL)	0.282	1.000	1.000–1.000			
PET positive	0.021	2.425	1.144–5.138	0.112	1.969	0.854–4.540

*HR* hazard ratio, *CI* confidence interval, *HCV* hepatitis C virus, *ALT* alanine aminotransferase, *HCC* hepatocellular carcinoma, *AFP* alpha-fetoprotein, *AFP-L3 Lens culinaris* agglutinin-reactive alpha-fetoprotein, *DCP*, Des-gamma-carboxyprothrombin, *PET* positron emission tomography

Table 4 lists the results of univariate and multivariate analyses of the background variables associated with metastatic recurrence. Univariate analysis identified the factors of Child–Pugh classification, type IV collagen 7S level, tumor size, number of tumors, AFP level, AFP-L3 (> 15%) level, and ^18^F-FDG-PET positivity as being significantly associated with metastatic recurrence after RFA. In multivariate analysis, ^18^F-FDG-PET positivity and tumor size were identified as independent factors.

**Table 4:** Univariate and multivariate analyses of factors related to metastatic recurrence

	Univariate analysis			Multivariate analysis		
	*p* value	HR	95%CI	*p* value	HR	95%CI
Age (years)	0.619	0.989	0.949–1.032			
Sex (female)	0.592	0.818	0.391–1.708			
Etiology (HCV)	0.631	1.246	0.508–3.052			
Child–Pugh class (A)	0.027	0.426	0.200–0.906	0.087	0.449	0.180–1.123
Platelets (×10^4^/μL)	0.588	1.014	0.965–1.064			
ALT (IU/L)	0.367	1.005	0.994–1.015			
Hyaluronic acid (ng/mL)	0.145	1.001	1.000–1.001			
Type IV collagen 7S (ng/mL)	0.018	1.141	1.023–1.274	0.454	1.057	0.914–1.222
Tumor size (mm)	0.000	1.132	1.062–1.207	0.017	1.089	1.015–1.169
Number of tumors	0.014	1.940	1.145–3.285	0.197	1.492	0.812–2.740
AFP (ng/mL)	0.012	1.001	1.000–1.002	0.534	1.000	0.998–1.001
AFP-L3 (> 15%)	0.030	2.559	1.096–5.972	0.329	1.703	0.585–4.961
DCP (mAU/mL)	0.775	1.000	1.000–1.000			
PET positive	< 0.001	12.941	4.646–36.047	< 0.001	10.297	3.128–33.898

*HR* hazard ratio, *CI* confidence interval, *HCV* hepatitis C virus, *ALT* alanine aminotransferase, *HCC* hepatocellular carcinoma, *AFP* alpha-fetoprotein, *AFP-L3*, *Lens culinaris* agglutinin-reactive alpha-fetoprotein, *DCP* Des-gamma-carboxyprothrombin, *PET* positron emission tomography

### Univariate and multivariate analyses of factors related to survival

Table 5 lists the results of univariate and multivariate analyses of background variables associated with survival. Univariate analysis identified the factors of tumor size, number of tumors, AFP level, AFP-L3 (> 15%) level, and ^18^F-FDG-PET positivity as being significantly associated with survival after RFA. In multivariate analysis, ^18^F-FDG-PET positivity and tumor size were identified as independent factors.

**Table 5:** Univariate and multivariate analyses of factors related to survival

	Univariate analysis			Multivariate analysis		
	*p* value	HR	95%CI	*p* value	HR	95%CI
Age (years)	0.648	1.014	0.955–1.076			
Sex (female)	0.253	0.517	0.167–1.603			
Etiology (HCV)	0.963	0.974	0.313–3.025			
Child–Pugh class (A)	0.147	0.456	0.158–1.316			
Platelets (×10^4^/μL)	0.139	1.033	0.990–1.078			
ALT (IU/L)	0.365	1.006	0.993–1.020			
Hyaluronic acid (ng/mL)	0.681	1.000	0.999–1.002			
Type IV collagen 7S (ng/mL)	0.070	1.150	0.989–1.338			
Tumor size (mm)	0.001	1.174	1.071–1.287	0.044	1.112	1.003–1.232
Number of tumors	0.037	2.035	1.044–3.965	0.535	1.315	0.554–3.122
AFP (ng/mL)	0.003	1.002	1.001–1.003	0.436	0.999	0.998–1.001
AFP-L3 (> 15%)	0.007	4.274	1.474–12.390	0.074	3.774	0.879–16.210
DCP (mAU/mL)	0.698	1.000	1.000–1.000			
PET positive	< 0.001	12.783	4.456–36.671	0.004	7.300	1.920–27.751

*HR* hazard ratio, *CI* confidence interval, *HCV* hepatitis C virus, *ALT* alanine aminotransferase, *HCC* hepatocellular carcinoma, *AFP* alpha-fetoprotein, *AFP-L3 Lens culinaris* agglutinin-reactive alpha-fetoprotein, *DCP* Des-gamma-carboxyprothrombin, *PET* positron emission tomography

## Discussion

To the best of our knowledge, the present study is the first to evaluate the prognostic value of ^18^F-FDG-PET in patients with small HCC treated by RFA. Although some studies have reported the prognostic value of ^18^F-FDG uptake in HCC patients who underwent liver resection or liver transplantation, most of these studies were retrospective [23]. Many previous studies have used visual analysis, standardized uptake value (SUV), and tumor-to-nontumor liver uptake ratio (TLR) as parameters for evaluating ^18^F-FDG uptake, and showed that ^18^F-FDG-PET can predict the risk of early recurrence or poor survival after surgical resection or liver transplantation. For example, Hatano et al. reported that overall survival after resection was significantly longer in the lower SUV ratio group (SUV ratio < 2) than in the higher SUV ratio group (SUV ratio > 2) [18]. Seo et al. also reported that overall and disease-free survival rates were significantly lower in the high SUV or TLR group than in the low SUV or TLR group [19]. Hyun et al. reported that higher TLR (≥2) was significantly associated with death in patients underwent curative treatments including resection, liver transplantation, and radiofrequency ablation [24]. Lim et al. showed that visual positivity of ^18^F-FDG-PET was an independent predictor for early recurrence after liver resection in their prospective observation study [25]. In the present study, visual qualitative analysis was used to evaluate ^18^F-FDG uptake because in clinical practice, visual analysis is easier than quantitative analysis. Furthermore, as SUV is a relative value that is influenced by the imaging conditions, there is no accepted optimal cut-off value and most of the currently published data on SUVs in tumors are of little or no value to investigators outside the laboratory where the investigation was conducted [26]. In this study, there was no significant correlation between recurrence and visual ^18^F-FDG-PET positivity. It is known that HCCs have several recurrence patterns such as local or multicentric or intrahepatic or extrahepatic metastasis. Especially multicentric recurrence is not affected by tumor factors such as visual ^18^F-FDG-PET positivity. Several kinds of recurrence were also included in this study, so visual ^18^F-FDG-PET positivity may not have been related to recurrence including all recurrence patterns. The present study found a significant association of visual ^18^F-FDG-PET positivity with early metastatic recurrence and survival after RFA. Metastatic recurrence is difficult to treat curatively, and leads to cancer death. The poor survival of ^18^F-FDG-PET positive patients with small HCC treated by RFA is probably attributable to the high-grade malignant potential of the HCC.

The risk of recurrence after ablation is related to the tumor characteristics at the time of therapy, which include size, degree of differentiation, and the presence or absence of lymphovascular invasion [17]. In the present study, tumor size and ^18^F-FDG-PET positivity were independent factors related to metastatic recurrence after RFA. Some reports have already indicated that ^18^F-FDG-PET has high predictive value as a surrogate for the presence of microvascular invasion [27]. Ochi et al. identified SUV max as the only independent predictive factor for microsatellite distance > 1 cm from the primary tumor lesion [28]. It would be difficult to achieve a complete cure by RFA in ^18^F-FDG-PET positive HCCs because these tumors are already locally advanced, with microvascular invasion and intrahepatic metastasis. Therefore, even though ^18^F-FDG-PET positive HCCs are within the indications for RFA, these tumors should not be treated by RFA as the first-line treatment. Park et al. found that compared with a surgical margin of < 1 cm, a surgical margin of > 1 cm significantly improved overall survival in ^18^F-FDG-PET positive patients but not in ^18^F-FDG-PET negative patients [29]. Accordingly, segmental hepatectomy with a sufficient surgical margin, or positron beam therapy that can obtain a wider safety margin than RFA, could be considered as first-line treatment. However, the outcome of hepatectomy for ^18^F-FDG-PET positive HCC is also poor. Further investigation is required to clarify whether a margin of > 1 cm is sufficient to improve the prognosis of patients with ^18^F-FDG-PET positive HCC. Combination therapy with RFA and transcatheter arterial chemoembolization or adjuvant therapy could also be considered; however, the routine use of adjuvant therapy for patients with HCC following successful resection or ablation is not recommended [17]. Further study is needed to determine the optimal treatment strategy for ^18^F-FDG-PET positive small HCC.

Several limitations must be considered when interpreting the results of the present study. First, the sample size is small because of the low proportion of ^18^F-FDG-PET positive HCCs among small HCCs. Especially, the proportion of ^18^F-FDG-PET positive HCCs was lower compared to ^18^F-FDG-PET negative HCCs which would have affected the statistical power. Therefore, a larger scale study is needed to validate our results. Second, histological evaluation of HCC was not performed. Intrahepatic cholangiocarcinoma (ICC) is also positive on ^18^F-FDG-PET, and we were unable to completely exclude ICC or combined ICC and HCC. Third, the optimal treatment strategy for ^18^F-FDG-PET positive small HCC cannot be derived from our results.

## Conclusions

In conclusion, ^18^F-FDG-PET positivity was significantly associated with outcomes after RFA. As RFA for ^18^F-FDG-PET positive small HCC has a high risk of metastatic recurrence and poor prognosis, RFA should not be readily selected as the first-line treatment, even though it is within the indications for RFA. ^18^F-FDG-PET should be performed in considering the optimal treatment strategy for small hypervascular HCCs diagnosed by contrast CT or MRI.

## Data Availability

The datasets used and/or analyzed during the current study are available from the corresponding author on reasonable request. However, there is no additional data available.

## Abbreviations

^18^F-FDG: ^18^F-fluorodeoxyglucose
HCC: Hepatocellular carcinoma
RFA: Radiofrequency ablation
PET: Positron emission tomography
CT: Computed tomography
MRI: Magnetic resonance imaging
ALT: Alanine aminotransferase
AFP: Alpha-fetoprotein
AFP-L3: *Lens culinaris* agglutinin-reactive alpha-fetoprotein
DCP: Des-gamma-carboxy prothrombin
HRs: Hazard ratios
CIs: Confidence intervals
HCV: Hepatitis C virus
SUV: Standardized uptake value
TLR: Tumor-to-nontumor liver uptake ratio
ICC: Intrahepatic cholangiocarcinoma
